# Diagnostic Value of Serum Angiogenesis Markers in Ovarian Cancer Using Multiplex Immunoassay

**DOI:** 10.3390/ijms18010123

**Published:** 2017-01-10

**Authors:** Agnieszka Horala, Agata Swiatly, Jan Matysiak, Paulina Banach, Ewa Nowak-Markwitz, Zenon J. Kokot

**Affiliations:** 1 Gynecologic Oncology Department, Poznan University of Medical Sciences, Polna 33, 60-535 Poznań, Poland; agnieszka0lemanska@gmail.com (A.H.); paulina@gmx.ch (P.B.); ewamarkwitz@poczta.fm (E.N.-M.); 2 Department of Inorganic and Analytical Chemistry, Poznan University of Medical Sciences, Grunwaldzka 6, 60-780 Poznań, Poland; agataswiatly@gmail.com (A.S.); jmatysiak@ump.edu.pl (J.M.)

**Keywords:** ovarian cancer, biomarkers, angiogenesis

## Abstract

As cancer development involves pathological vessel formation, 16 angiogenesis markers were evaluated as potential ovarian cancer (OC) biomarkers. Blood samples collected from 172 patients were divided based on histopathological result: OC (*n* = 38), borderline ovarian tumours (*n* = 6), non-malignant ovarian tumours (*n* = 62), healthy controls (*n* = 50) and 16 patients were excluded. Sixteen angiogenesis markers were measured using BioPlex Pro Human Cancer Biomarker Panel 1 immunoassay. Additionally, concentrations of cancer antigen 125 (CA125) and human epididymis protein 4 (HE4) were measured in patients with adnexal masses using electrochemiluminescence immunoassay. In the comparison between OC vs. non-OC, osteopontin achieved the highest area under the curve (AUC) of 0.79 (sensitivity 69%, specificity 78%). Multimarker models based on four to six markers (basic fibroblast growth factor—FGF-basic, follistatin, hepatocyte growth factor—HGF, osteopontin, platelet-derived growth factor AB/BB—PDGF-AB/BB, leptin) demonstrated higher discriminatory ability (AUC 0.80–0.81) than a single marker (AUC 0.79). When comparing OC with benign ovarian tumours, six markers had statistically different expression (osteopontin, leptin, follistatin, PDGF-AB/BB, HGF, FGF-basic). Osteopontin was the best single angiogenesis marker (AUC 0.825, sensitivity 72%, specificity 82%). A three-marker panel consisting of osteopontin, CA125 and HE4 better discriminated the groups (AUC 0.958) than HE4 or CA125 alone (AUC 0.941 and 0.932, respectively). Osteopontin should be further investigated as a potential biomarker in OC screening and differential diagnosis of ovarian tumours. Adding osteopontin to a panel of already used biomarkers (CA125 and HE4) significantly improves differential diagnosis between malignant and benign ovarian tumours.

## 1. Introduction

Ovarian cancer (OC) remains the most deadly gynaecological malignancy and is responsible for 4.3% of deaths due to neoplasms in women worldwide and 6.1% in Poland [1]. Due to the scarceness and low specificity of early symptoms, this malignancy often develops undetected until reaching advanced stages, when the prognosis is poor and the treatment options are limited. When diagnosed at an early stage, ovarian cancer can be curable with conventional surgery and chemotherapy in up to 90% of cases. Five-year survival rates in stage I disease (according to classification by International Federation of Gynaecology and Obstetrics (FIGO)) range from 83.4%–89.6% [2]. Unfortunately, at present only about 25% of women are diagnosed at FIGO I–II stage, while in over 70% the disease is advanced (FIGO stage III–IV) at the moment of diagnosis when five-year survival drops to 30% [2].

No effective OC screening markers exist and there is no country with an efficient screening programme. The diagnosis of an ovarian tumour is based on clinical assessment (risk factors, symptoms and physical examination) followed by transvaginal ultrasound examination. Both methods are highly subjective, have low specificity and are not recommended in screening [3]. On transvaginal ultrasound benign and malignant adnexal masses can be distinguished with a sensitivity and specificity of 86%–94% and 94%–96%, respectively [3]. In recent years, two biomarkers, cancer antigen 125 (CA125) and human epididymis protein 4 (HE4) have been introduced into clinical practice in the diagnostic process but their use is limited to patients with adnexal masses identified previously in transvaginal ultrasound. Correct differentiation between benign and malignant pathologies of the ovary, especially among premenopausal women, is often possible only by histological examination of the tissue. This results in unnecessary surgical procedures that could be avoided if a reliable non-invasive diagnostic method existed, as over 90% of ovarian masses detected in pre-menopausal women and up to 60% of those in post-menopausal women are benign [4].

When finding a universal OC biomarker did not seem feasible, in 2004 Kurman et al. [5] proposed a dualistic model of OC pathogenesis based on clinical and molecular analysis. The model divides OC type I (encompassing serous grade 1, endometrial grade 1, mucinous and clearcell OC) and type II (including serous grade 2–3, endometrial grade 2–3, carcinosarcoma and undifferentiated OC). Type I OC accounts for 25% of all OC cases, is usually diagnosed at earlier stages, tumor growth is slower and prognosis is better compared to type II ovarian cancer. This model was also addressed in the presented paper.

As the development of a neoplasm involves intensive formation of new blood vessels, angiogenesis biomarkers seem to be a feasible target to investigate. Since late 1960s many studies have shown that tumour cells produce diffusible factors which mediate tumour angiogenesis. It has become an established factor in human carcinogenesis that influences tumour growth and invasion [6]. Consequently, many angiogenesis markers have been identified. Identifying a specific disease indicator(s) in serum could provide a convenient and non-invasive diagnostic method, help to monitor the treatment or disease progression and possibly enable the development of new treatment strategies. Many studies investigated the expression of angiogenesis markers, alone or in small panels, in various malignancies [7,8,9,10,11,12] showing promising results. As a consequence, anti-angiogenic treatment has been extensively investigated and already introduced into clinical practice with significant therapeutic effect for several cancers, including OC [13]. 

To the best of our knowledge, this is the first study which uses this set of angiogenesis multi-marker Bioplex panel for OC study. Additionally, the studied groups encompassed not only healthy controls and OC patients, but also patients with benign ovarian tumours. Adoption of novel methodology enabled simultaneous determination of serum levels of 16 different angiogenesis markers in one experiment. The aim of this study was to evaluate the usefulness of angiogenesis markers in OC detection and differential diagnosis of ovarian tumours (Table 1). The utility of sets of various markers was examined in order to identify the most effective combination. Moreover, they were further compared with markers already used in clinical practice (CA125 and HE4) as a diagnostic tool of OC. 

## 2. Results

### 2.1. Usefulness of Angiogenesis Factors in Diagnosing Ovarian Cancer

The OC group was compared to the control group that consisted of patients with benign ovarian tumours and healthy controls (borderline tumours were not included in this analysis). In the OC group, circulating levels of five markers (basic fibroblast growth factor—FGF-basic, follistatin, hepatocyte growth factor—HGF, osteopontin and platelet-derived growth factor AB/BB—PDGF-AB/BB) were significantly increased (*p* < 0.03) in comparison to the control group, while the level of leptin was significantly decreased (*p* = 0.0014) (Table 1 and Table 2). Their discriminative ability was further checked by calculating the receiver operating characteristic (ROC) curves which give a graphical presentation of sensitivity and specificity of the studied factors. Areas under the curve (AUC) above 0.75 were considered to characterize a satisfactory discriminating factor. The highest obtained AUC value (0.79) was achieved by osteopontin with sensitivity of 69% and specificity of 78% at a cut-off value of 41,435.1 pg/mL (Table 2). 

The obtained results were also analysed using Partial-Least Squares Discriminant Analysis (PLS-DA) in order to distinguish the studied groups. This chemometric analysis confirmed that the same angiogenesis markers, that were selected earlier in univariate tests, have the best efficacy in discriminating between groups (Variable Importance in Projection—VIP score > 1.0). Two markers—osteopontin and follistatin—achieved the VIP scores above 1.5. According to univariate and multivariate analyses, osteopontin seems to be the best marker to distinguish patients with OC and control group (i.e., healthy individuals and patients with benign ovarian tumours). 

**Figure 1:**
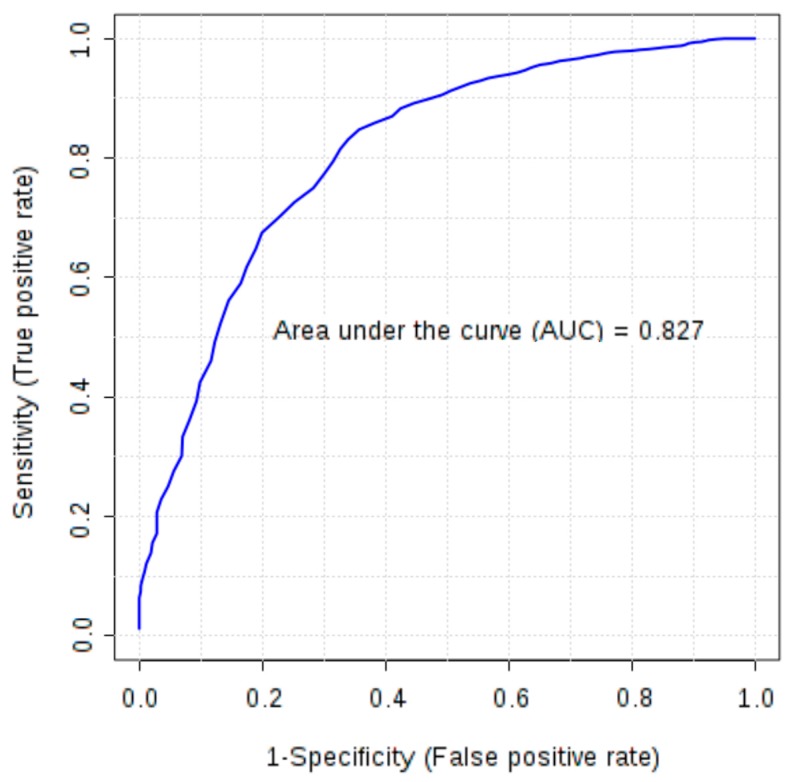
Multivariate receiver operating characteristic (ROC) curve representing correlation between serum concentrations of FGF-basic, follistatin, osteopontin and PDGF-AB/BB in ovarian cancer patients and control group (healthy subjects and patients with benign ovarian tumours).

Furthermore, combinations of six markers (FGF-basic, follistatin, HGF, osteopontin, PDGF-AB/BB and leptin), earlier selected as significant in univariate tests, were evaluated using multivariate ROC analysis. All created models were characterized by AUC above 0.77. Models based on four to six markers demonstrated higher discriminatory ability (AUC 0.80–0.81) than a single marker, osteopontin (AUC > 0.79). In the models based on four and five features osteopontin, PDGF-AB/BB, FGF-basic and follistatin were the most frequently used markers due to their high ability to differentiate studied groups and the AUC based only on these four markers was 0.827 (Figure 1). 

### 2.2. Usefulness of Angiogenesis Factors in Distinguishing Ovarian Cancer Types

In further analysis angiogenesis profiles of type I and type II OC were compared with healthy controls using *t*-test or Mann-Whitney test (Table 2). Due to growing evidence on similarities in pathogenesis of type I OC and borderline ovarian tumours [5] they were included in the analysis as type I OC. This group was characterized by decreased level of sHER2/neu (*p* = 0.035). According to the ROC curve this angiogenesis factor distinguishes type I OC patients and healthy controls with a sensitivity of 75% and specificity of 65% and reaches the AUC of 0.70 (cut off concentration: 3854.14 pg/mL). The VIP score for sHER2/neu was 1.76. 

The comparison of the angiogenesis panel between patients with type II OC and healthy controls revealed significantly higher concentrations of FGF-basic, follistatin, G-CSF, HGF, osteopontin, PDGF-AB/BB and lower levels of leptin in the type II OC group. The AUC values for these markers were above 0.64. The ROC curve for osteopontin with cut-off concentration at 41,020.7 pg/mL discriminated the studied groups with a sensitivity of 66.7% and specificity of 82.8%, while the AUC was 0.82. The VIP score for osteopontin was 2.38 which is in agreement with the results obtained by univariate statistical tests.

### 2.3. Usefulness of Angiogenesis Factors in Differential Diagnosis of Ovarian Tumours

In order to distinguish ovarian cancer from benign ovarian tumours, the serum concentration of angiogenesis markers in those groups were compared (borderline tumours were excluded from this analysis). Six markers had statistically different expression (osteopontin, leptin, follistatin, PDGF-AB/BB, HGF, FGF-basic) (Table 2). In the ROC analysis osteopontin obtained the highest AUC among all proteins (AUC 0.825) (Table 2) with a cut-off value of 45,300 pg/mL and sensitivity and specificity of 72% and 82%, respectively. The PLS-DA and VIP-score analysis led to a conclusion that three markers, osteopontin, follistatin, PDGF-AB/BB, were characterized by the highest discriminatory ability. Moreover, the multivariate ROC curve based on those three markers allowed to obtain the AUC of 0.819. 

**Figure 2:**
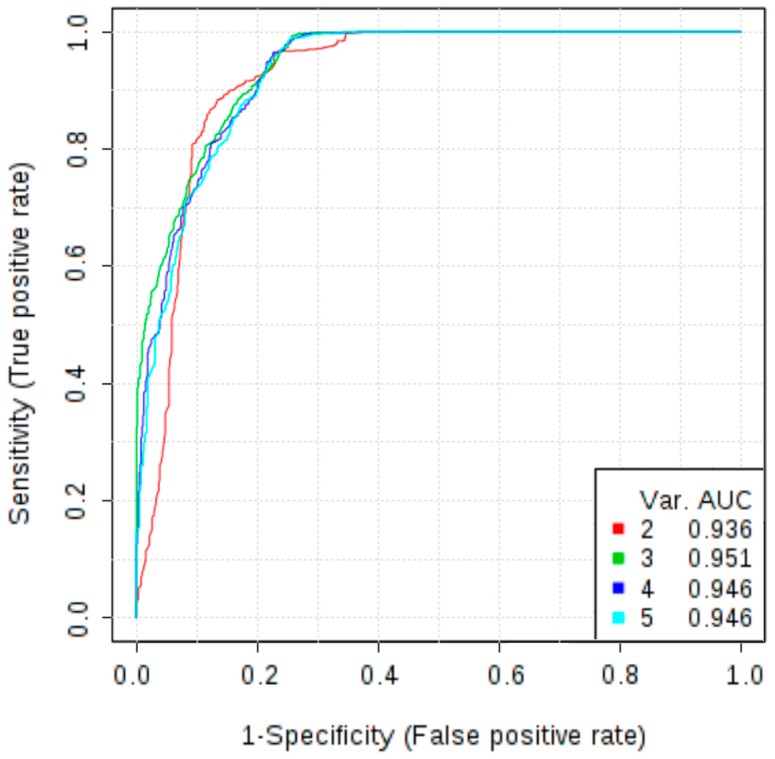
Multivariate receiver operating characteristic (ROC) curves representing correlations between concentrations of CA125, HE4, osteopontin, follistatin, PDGF-AB/BB in differentiating non-malignant ovarian tumours vs. ovarian cancer; Models **2** and **3** are mostly based on CA125, HE4 and osteopontin; Model **2** allowed to obtain an AUC of 0.936, while the model **3**, in which osteopontin is more frequently selected, increased the AUC to 0.951; The use of additional angiogenesis markers (models **4** and **5**) did not improve the diagnostic accuracy.

In addition to the potential novel biomarkers investigated in this study, we measured the concentrations of markers that are already used in clinical practice, CA125 and HE4. Wilcoxon test revealed that serum levels of HE4 and CA125 were significantly increased in patients with OC in comparison with patients with non-malignant ovarian tumour (*p* < 0.001). At a cut-off level of 87.625 pmol/L HE4 showed a sensitivity of 96.2% and specificity of 87.2%, while the AUC was 0.941. Analysis of the ROC curve of CA125 allowed to obtain a sensitivity of 90.6%, specificity of 84.6% and AUC of 0.932 at a cut-off level 71.365 U/mL. To further evaluate the role of osteopontin as a diagnostic test to discriminate malignant and non-malignant ovarian tumours, a multivariate ROC curve analysis was performed. The combined model of three markers: osteopontin, CA125 and HE4 reached AUC of 0.958. Therefore, adding osteopontin to the panel of markers improved diagnostic accuracy as compared to the model based only on HE4 and CA125 (which AUC was 0.943). The addition of other markers does not increase the AUC value (Figure 2). 

## 3. Discussion

As angiogenesis is a key process in neoplasm formation, extensive research has been ongoing to assess the usefulness of its markers in diagnosis and treatment of various cancers, e.g., breast [11,14], colorectal [10], pancreatic [7] and lung cancer [12]. Moreover, the substances involved in angiogenesis have become promising therapeutic targets, e.g., bevacizumab (anti-VEGF monoclonal antibody) is already used in clinical practice in combination with standard chemotherapy to treat ovarian, cervical, colorectal, nonsquamous non-small cell (NSCLC) lung, kidney and brain cancers [13].

One of the popular methods in biomarker studies is enzyme-linked immunosorbent assay (ELISA). However, this strategy is expensive, time-consuming and requires large sample volume. Another problem in study design on biological markers is the limited number of substances that can be tested simultaneously. Therefore, a microarray analysis has been proposed to determine levels of 169 proteins in serum samples from healthy individuals, newly diagnosed women with OC and patients with recurrent disease [15]. However, obtained results of median fluorescence intensity still had to be confirmed with fully quantitative ELISA assays. Therefore, in our study simultaneous measurement of multiple angiogenesis serum markers based on Bio-Plex technology (Bio-Rad) was proposed. This strategy enabled the determination of particular proteins in one assay and made this a unique angiogenesis multi-marker OC study. Another strength of this analysis is the inclusion of not only healthy controls and OC patients but also patients with benign ovarian tumours. Additionally, the concentrations of CA125 and HE4 were measured using another method: electro-chemiluminescence immunoassay (ECLIA). This technique is routinely used in many laboratories and hospitals in ovarian cancer studies [16,17]. According to rigorous validation procedures made by manufacturers, the measured concentrations obtained in both Bioplex and ECLIA analysis are reliable and they can be compared in statistical analysis to evaluate the usefulness of the studied angiogenesis panel. 

In our study, we evaluated the usefulness of 16 markers in OC diagnostics: soluble epidermal growth factor receptor—sEGFR, basic fibroblast growth factor—FGF-basic, follistatin, granulocyte colony-stimulating factor—G-CSF, hepatocyte growth factor —HGF, soluble human epidermal growth factor receptor 2—sHER2/neu, soluble interleukin 6—sIL-6Rα, leptin, osteopontin, platelet and endothelial cell adhesion molecule 1—PECAM-1, platelet-derived growth factor AB/BB—PDGF-AB/BB, prolactin, stem cell factor—SCF, soluble receptor tyrosine kinase 2—sTIE-2, soluble vascular endothelial growth factor receptor 1 and 2—sVEGFR-1 and sVEGFR-2. For eight of these markers, targeted therapies are already under evaluation in clinical trials (Table 3) [18,19,20,21,22,23,24] and some of the targeted drugs are already approved for clinical use in other than OC malignancies [23]. 

Our research confirmed that serum concentrations of analysed angiogenesis markers vary between the studied groups. The analysis that compared OC patients vs. benign ovarian tumours and healthy controls was performed in order to search for a marker useful in screening. Osteopontin was identified as the best single marker that allowed to distinguish those groups (Table 2). Creating multi-marker panels only slightly improved the diagnostic accuracy. Differences in the expression of angiogenesis markers between type I and type II of OC were also evaluated. The serum level of only one marker—sHER2/neu—was significantly different when comparing OC type I with healthy controls while seven markers significantly distinguished type II OC from healthy controls (FGF-basic, follistatin, G-CSF, HGF, leptin, osteopontin, PDGF-AB/BB) (Table 2). This leads to a conclusion that angiogenesis is more pronounced in OC type II and can be indicative of its higher aggressiveness and more rapid spread. Moreover, it undermines the significance of those markers in diagnosing type I OC and supports Kurman’s theory about substantial discrepancies between OC types that should be treated as separate entities [5]. 

Further analysis focused on differential diagnosis of ovarian tumours. The OC group was compared with benign ovarian tumour group. Expression of six markers differed significantly and, what needs to be emphasized, all those markers proved also to be significantly differently expressed in diagnosing OC and distinguishing OC types. Osteopontin was proved to be the best single angiogenesis marker to distinguish malignant and benign ovarian tumours with *p*-value below 0.001 and AUC equal 0.825. The concentrations of markers already used in clinical practice, CA125 and HE4 were also measured. Both markers differentiated studied groups significantly (Table 2). However, adding osteopontin to a multi-marker panel significantly improved the accuracy of the diagnosis (AUC 0.958), thus asserting its potential role as OC marker.

Osteopontin was discovered as a protein secreted by osteoblasts in bone, although it is also expressed in many other tissues and is involved in the number of different signalling pathways, such as inflammation, immune response or angiogenesis [25]. Its levels were reported to be elevated in several malignancies such as: breast, cervical, colorectal, prostate, lung and pancreatic cancers [26]. In OC, it was proved to promote cancer cell growth, migration and invasion [27] and to be an independent predictor of poor prognosis [28].

Our results, pointing at osteopontin as the most efficient of the 16 investigated angiogenesis markers, are consistent with the results of meta-analyses that confirmed its elevated levels in OC and concluded that it could be useful in diagnosing OC [29,30]. Another meta-analysis showed its usefulness as an adjunct to CA125. It reported an overall diagnostic sensitivity and specificity of ostepontin as a single marker in OC of 76.6% and 89.7%, respectively, while a combined test with CA125 showed the sensitivity and specificity of 87.1% and 88.1%, respectively [31], which corresponds with our results (Figure 2).

Another discriminatory marker in our study was follistatin. Its primary function is binding and neutralization of activin, a paracrine hormone, which is often elevated in OC and therefore is a potential therapeutic target in this malignancy [32]. It was also reported as a marker for endometriosis [33]. In a study by Ren et al. on 245 patients, including 45 with OC, follistatin was found to be significantly elevated in patients with OC compared with healthy individuals [34], which is in agreement with our findings (Table 1 and Table 2).

Platelet-derived growth factor (PDGF), which in our study was elevated in OC patients, plays a role in cell growth, chemotaxis, development of a vascular connective tissue stroma in tumorigenesis as well as may contribute to lymphatic metastases or be involved in the tumour evasion of the anti-VEGF treatment [35,36]. Many malignant tumours are characterised by high expression of PDGF ligands and/or receptors, including OC and it is thought to be related to OC development and progression [37]. Two targeted drugs, imatinib and sunitinib, are registered for clinical use in treatment for leukaemia, GIST (gastrointestinal stromal tumours), renal and hepatocellular cancers [23] and more recently, rapid development of targeted therapies against PDGF pathway in OC have been investigated [38]. In a study by Madsen et al. [39], median PDGF-AA, PDGF-BB and FGF2 levels were significantly elevated in patients with OC compared to borderline tumours, normal ovaries or benign tumours. This study also reported an association between preoperative serum PDGF-AA and PDGF-BB levels and FIGO stage and residual tumour after surgery in patients with OC indicating its possible application as an indicator of radicality of cytoreductive surgery. Additionally, high serum concentrations of PDGF-BB and FGF2 were also proved to have prognostic significance in patients with recurrent OC treated with bevacizumab [40]. Our results confirmed that PDGF expression is elevated in OC patients (Table 1 and Table 2) and can be useful in diagnosing this disease, especially as a component of a multi-marker model.

In our analysis, FGF2 was overexpressed in OC group (Table 1 and Table 2). FGF2 is a growth factor with a strong pro-angiogenic function, affecting endothelial cell migration and proliferation [41]. Increased serum levels of FGF2 were found in multiple malignancies, including breast [11], pancreatic, non-small cell lung and prostate cancers [41]. Our results are consistent with other studies that reported elevated FGF2 serum concentrations in patients with epithelial OC [39,42] although the study by Madsen et al. [39] investigating three angiogenesis markers from our panel (PDGF-AA, PDGF-BB, FGF2) did not consider them useful for diagnostic purposes due to high degree of overlapping values among different patient groups.

Consistently with our results, significantly higher preoperative HGF serum levels in OC patients compared with benign and borderline ovarian tumours were also demonstrated in a study by Aune et al. [43]. This study additionally showed a negative correlation between preoperative HGF level and disease-free survival. The marker was also proved to enhance cell invasion [44] and stimulate peritoneal implantation in OC [45]. 

The data regarding leptin expression in serum of OC patients is inconsistent. It is principally a product of adipose tissue, nevertheless its expression was detected in other tissues including cancer cells, where its involvement in carcinogenesis by paracrine and autocrine mechanisms was described [46]. There are some studies reporting its increased levels, however the majority of authors report decreased levels of leptin in OC compared to healthy individuals [15,46], as observed in our study. Contradictory to those findings, elevated leptin levels stimulated OC cell migration and invasion and were associated with poor prognosis in obese women [47].

What may seem surprising is the fact that two studied markers, sVEGFR-1 and sVEGFR-2, did not show statistically significant differences in any of the analyses. An FDA (Food and Drug Administration)-approved drug used in treatment of various cancers, bevacizumab, is a recombinant humanized monoclonal antibody that specifically binds vascular endothelial growth factor (VEGF) preventing receptor binding and inhibiting vessel formation. A significant increase in serum VEGF levels in OC patients was shown in several studies [8,48] and VEGF overexpression has been associated with metastasis formation and poor prognosis [48]. However, VEGF receptors are expressed principally on the surface of endothelial cells of blood (VEGFR-1, VEGFR-2) and lymphatic vessels (VEGFR-3) [49] and while VEGF was found to be elevated in serum, VEGFRs were demonstrated to be overexpressed in OC tissue [50]. In a study by Spannuth et al., VEGFR-2 was overexpressed in 85% of human OC specimens and VEGFR-1 in 15% [50]. This explains why in our study a correlation between serum levels of VEGFR-1 and VEGFR-2 and OC was not observed although several targeted therapies against VEGFRs underwent evaluation in randomised controlled trials showing promising results (an increase in progression-free survival–PFS but no overall survival–OS improvement was seen in meta-analysis of six randomised controlled trials) [51]. 

## 4. Methods

### 4.1. Patient Characteristics

The study was conducted in accordance with the Declaration of Helsinki and the protocol was approved by the local Bioethical Commission of Poznan University of Medical Sciences, Poland (Decision No. 165/16). A written consent for inclusion was obtained from all participants prior to sample collection. Blood samples were collected from 172 patients operated in Gynecologic Oncology Department on the day before the surgery between August 2014 and December 2015. Blood samples were incubated for 30 min in room temperature for clotting, then centrifuged for 15 min at 4000 rpm. Serum was isolated and stored at −80 °C until analysis. Sixteen patients who met exclusion criteria (any other malignancy currently or in anamnesis and ovarian malignancy other than epithelial OC) were disqualified and 156 patients were included in the final analysis. Based on the histopathological result the patients were then divided into 4 groups: OC (38 patients), non-malignant ovarian tumours (62 patients), no pathology of the ovaries (further referred to as “healthy controls”) (50 patients) and borderline ovarian tumours (6 patients). Additionally, different types of OC were identified: type I OC (7 OC patients and 6 borderline tumour patients) and type II OC (31 patients) according to the clinicopathological classification proposed by Kurman et al. [5]. Study group characteristics are presented in Table 4 and Table 5. Borderline tumours are a heterogeneous group characterized by atypical epithelial proliferation without stromal invasion. Although occasionally they give implants in the omentum and peritoneum, they are considered a distinct clinical entity than ovarian cancer. The usefulness of CA125 and HE4 in borderline tumour detection is unclear [52,53,54]. On the other hand, borderline ovarian tumours share the staging system with invasive ovarian cancer and have a significant risk of recurrence after conservative surgery. In patients with advanced-stage disease the risk of progression to invasive disease is clinically significant. The rate of invasive recurrences reached up to 6% in some reports [55] although the risk of malignant transformation is unclear. For those reasons and because of the limited number of samples (*n* = 6) we decided to exclude borderline tumours from the analyses comparing ovarian cancer against healthy controls and benign tumours. However, taking into account the increasing evidence that the pathogenesis of borderline ovarian tumours and low-grade serous carcinomas (type I OC) involves similar genes and pathways that are distinct from those identified in high-grade serous carcinomas (type II OC) [5], we decided to include borderline tumours in the type I OC group compared against type II OC group. 

CA125 and HE4 serum concentrations were quantitatively measured in patients with adnexal masses by electrochemiluminescence immunoassay on Roche Cobas System (Roche Diagnostics, Indianapolis, IN, USA) in the Central Hospital Laboratory according to the manufacturer’s instructions. This analysis uses biotinylated and ruthenylated monoclonal antibodies against HE4 and CA125 which form complex with streptavidin microparticules. In order to precisely control specificity of the chemiluminescence reaction, it is induced by applying a voltage on a sample solution which enables the detection of the reaction complex. The standard cut-off values are 35 U/mL for CA125 and 140 pmol/L for HE4, however for the purpose of this study optimal cut-off levels were identified. 

### 4.2. Measurement of Angiogenesis Panel

Using 96-well-plate bead-based immunoassay (Bio-Plex Pro Human Cancer Biomarker Panel 1, Bio-Rad, Hercules, CA, USA), quantification of serum concentrations of angiogenesis markers was performed. The angiogenesis panel was composed of 16 markers: sEGFR, FGF-basic, follistatin, G-CSF, HGF, sHER2/neu, sIL-6Rα, leptin, osteopontin, PECAM-1, PDGF-AB/BB, prolactin, SCF, sTIE-2, sVEGFR-1 and sVEGFR-2. The analysis was performed according to the manufacturer’s instructions. In brief, 50 μL of serum samples, standards and quality controls were added to wells containing the antibody-coupled beads. After the incubation period and washing, detection antibody-biotin reporters were added to each well and incubated. The last step was incubation of the beads with the fluorescent conjugate streptavidin-phycoerythrin. The concentrations were measured using Bio-Plex array reader (Bio-Plex MAGPIX, Bio-Rad, Hercules, CA, USA) based on flow cytometry. Data acquisition was determined by the Bio-Plex Manager 6.0 software. Calibration and verification were performed before the analysis. The standard curves were optimized automatically. The concentrations of analyzed markers were expressed as picograms per milliliter (pg/mL) according to the standard curves. Blanks containing only manufacturer’s diluents and high and low quality controls and were analyzed in each assay in duplicate. Table 1 contains average levels and standard deviation (SD) of studied markers in tested groups. 

### 4.3. Data Analysis

Univariate data analysis including Shapiro-Wilk test, *t*-test and Mann-Whitney test were performed using Statistica software (version 12.0; StatSoft Inc., Tulsa, OK, USA). Comparison between studied groups were evaluated with *t*-test or Mann-Whitney test depending on the mode of distribution. *p*-values < 0.05 were considered to be statistically significant. Data distribution was tested with Shapiro-Wilk test. Univariate and multivariate receiver operating characteristic (ROC) curve and Partial-Least Squares Discriminant Analysis (PLS-DA) were calculated by MetaboAnalyst 3.0 web portal. For each analyte classical univariate (ROC) curve was evaluated to show graphical correlation between specificity and sensitivity. Moreover, for selection and classification of the most relevant features PLS-DA was applied. PLS-DA is a standard supervised chemometric analysis that uses multiple linear regression model to provide linear relations between multivariate measurements. Variable Importance in Projection (VIP) scores estimate the variable’s importance in the PLS-DA model [56]. The higher the VIP score, the more important is the studied variable in the classification. This method allowed us to select the most relevant angiogenesis factors in multimarker models. Multivariate receiver operating characteristic (ROC) curve based on support vector machines (SVM) was calculated for features with the highest discriminative ability. In order to make the data obtained by different techniques (ECLIA or Bioplex) comparable, it was pre-processed using the scaling option in Metaboanalyst web portal. 

## 5. Conclusions

In this study, we investigated the diagnostic usefulness of 16 angiogenesis markers in patients with epithelial OC. The application of a novel immunoassay technique allowed to assess the diagnostic utility of a broad panel of substances involved in angiogenesis simultaneously. We confirmed that osteopontin has a strong potential as a marker in non-invasive diagnostics of OC in both screening and differential diagnosis of ovarian tumours. We identified other, less extensively investigated angiogenesis markers that could be helpful in OC diagnosis and/or serve as therapeutic targets. Furthermore, we showed that adding osteopontin to a panel of used biomarkers (CA125 and HE4) significantly improves differential diagnosis between malignant and benign ovarian tumours. These findings clearly point at osteopontin as a promising target for further investigations. Obtained values of sensitivity and specificity for investigated markers are insufficient for wide population screening. However, panels involving those markers could be considered for non-invasive screening in high-risk populations such as patients with strong familial history of breast/ovarian cancer and with *BRCA1* or *BRCA2* (breast cancer gene 1 and 2, respectively) mutations where early and more accurate OC detection would facilitate the determination of the optimal time for preventive surgery. Our research can contribute to the improvement of OC diagnostic methods. Further and large-scale studies are needed to definitively prove the usefulness of the studied markers in clinical practice. Taking into account the growing interest in the angiogenesis process and promising preliminary results of targeted antiangiogenic therapies, further research is warranted. 

## Figures and Tables

**Table 1:** Serum average levels and standard deviation (SD) of 16 markers: sEGFR, FGF-basic, follistatin, G-CSF, HGF, sHER2/neu, sIL-6Rα, leptin, osteopontin, PECAM-1, PDGF-AB/BB, prolactin, SCF, sTIE-2, sVEGFR-1 and sVEGFR-2 in tested groups.

Angiogenesis Marker	Ovarian Cancer						Control Group					
	Type I		Type II		Total		Non-Malignant Tumor		Healthy Subjects		Total	
	Average	SD	Average	SD	Average	SD	Average	SD	Average	SD	Average	SD
sEGFR	20,342.13	5845.31	20,295.90	8421.43	20,309.43	20,309.43	21,309.90	6318.37	22,883.43	9007.22	22,009.25	7633.25
FGF-basic	180.48	44.22	203.77	63.91	196.95	196.95	179.00	52.12	180.85	52.60	179.82	52.10
Follistatin	685.78	317.57	1042.48	844.22	938.08	938.08	547.91	232.49	629.53	322.66	584.19	277.84
G-CSF	92.53	26.26	109.25	49.04	104.36	104.36	88.79	23.68	87.51	25.59	88.22	24.44
sHER2/neu	3320.85	948.04	4091.06	2061.63	3865.63	3865.63	3563.75	1273.51	4428.57	2414.32	3948.11	1908.16
HGF	1440.99	779.83	1936.05	1028.92	1791.16	1791.16	1305.13	544.67	1444.71	711.23	1367.17	625.01
sIL-6Rα	15,154.04	4441.99	17,100.30	9621.15	16,530.66	16,530.66	14,733.51	5632.62	17,920.81	12,282.34	16,150.09	9289.22
Leptin	7928.88	7133.65	5892.49	6349.54	6488.51	6488.51	11,162.45	9759.36	12,370.69	14,333.84	11,699.44	11,963.72
Osteopontin	46,882.40	19,429.79	73,250.94	34,155.80	65,533.32	65,533.32	34816.71	15,300.22	38,185.47	20,838.74	36,313.94	17,962.64
PDGF-AB/BB	4396.93	2274.18	5397.20	2941.45	5104.44	5104.44	3653.87	1517.89	4007.50	2120.66	3811.04	1810.25
PECAM-1	4411.84	996.33	4748.20	1674.27	4649.75	4649.75	4461.57	1524.40	4981.73	2088.27	4692.75	1806.74
Prolactin	11,688.28	9112.09	8920.90	5128.69	9730.87	9730.87	13,006.48	14,247.64	8799.15	6513.54	11,136.55	11,618.07
SCF	183.67	56.25	201.16	84.71	196.04	196.04	191.02	64.48	208.57	108.36	198.82	86.76
sTIE-2	7697.06	2707.86	8890.87	4578.14	8541.46	8541.46	7824.73	2841.97	7886.31	3240.18	7852.10	3011.00
sVEGFR-1	323.51	200.50	373.64	237.94	358.96	358.96	288.61	122.69	292.56	150.46	290.37	135.08
sVEGFR-2	2906.88	1024.60	3347.97	1631.41	3218.87	3218.87	3128.36	1127.17	3520.94	1827.38	3302.84	1485.19
CA125	344.68	536.27	1053.88	1312.09	853.85	1184.66	44.75	82.41	-	-	-	-
HE4	258.78	399.07	760.70	942.74	619.14	851.91	50.22	13.88	-	-	-	-

sEGFR: soluble epidermal growth factor receptor; FGF-basic: basic fibroblast growth factor; G-CSF: granulocyte colony-stimulating factor; sHER2/neu:soluble human epidermal growth factor receptor 2; HGF: hepatocyte growth factor; sIL-6Rα: soluble interleukin 6; PDGF-AB/BB: platelet-derived growth factor AB/BB; PECAM-1: platelet and endothelial cell adhesion molecule 1; SCF: stem cell factor; sTIE-2: soluble receptor tyrosine kinase; sVEGFR: soluble vascular endothelial growth factor receptor; CA125: cancer antigen 125; HE4: human epididymis protein 4; SD: standard deviation.

**Table 2:** Discriminatory value of serum angiogenesis markers expression showing significant *p*-values (*p* < 0.05) and area under the receiver operating characteristic (ROC) curve (AUC > 0.610) between studied groups.

Angiogenesis Marker	OC vs. Control Group		Type I OC vs. Healthy Controls		Type II OC vs. Healthy Controls		OC vs. Benign Ovarian Tumours	
	*p*-Value	AUC	*p*-Value	AUC	*p*-Value	AUC	*p*-Value	AUC
FGF-basic	0.0288	0.617	-	-	0.035	0.642	0.026	0.636
Follistatin	0.002	0.668	-	-	0.013	0.675	<0.001	0.713
G-CSF	-	-	-	-	0.048	0.643	-	-
sHER2/neu	-	-	0.035	0.704	-	-	-	-
HGF	0.02	0.619	-	-	0.036	0.645	0.020	0.643
Leptin	0.001	0.669	-	-	0.005	0.696	<0.001	0.715
Osteopontin	<0.001	**0.791**	-	-	<0.001	0.82	<0.001	**0.825**
PDGF-AB/BB	0.008	0.636	-	-	0.019	0.645	0.001	0.652
CA125	-	-	-	-	-	-	<0.001	0.935
HE4	-	-	-	-	-	-	<0.001	0.946

The highest obtained AUC values are bolded; OC: ovarian cancer; AUC: area under the curve.

**Table 3:** Examples of the targeted therapies which are already under evaluation in clinical trials for eight studied markers.

Marker	Full Name	Target Drugs	Citation
sEGFR	soluble epidermal growth factor receptor	Gefitinib; Erlotinib; Cetuximab	Murphy et al. [18]; Secord et al. [19]
sHER-2/neu	human epidermal growth factor receptor 2 erbB-2, ERBB2	Trastuzumab (Herceptin)	Ray-Coquard et al. [20]
HGF	hepatocyte growth factor	Rilotumumab	Martin et al. [21]
FGF-basic	basic fibroblast growth factor	Nintedanib (VEGFR, PDGFR, FGFR inhibitor); Pazopanib (VEGFR, PDGFR, FGFR inhibitor); Lucitanib (VEGFR 1–3 and FGFR 1–2 inhibitor)	Ivy et al. [22]
PDGF-AB/BB	platelet-derived growth factor—a dimeric glycoprotein composed of two A (-AA) or two B (-BB) chains or a combination of the two (-AB)	Cediranib (VEGFR 1–3, PDGFR inhibitor); Sorafenib (VEGFR, PDGFR inhibitor); Sunitinib (VEGFR, PDGFR, SCF inhibitor); Nintedanib (VEGFR, PDGFR, FGFR inhibitor); Pazopanib (VEGFR, PDGFR, FGFR inhibitor); Imatinib (PDGFRs and SCF inhibitor)	Ivy et al. [22]; Choi et al. [23]
sVEGFR-1 (sVEGFR1/sFLT1)	soluble vascular endothelial growth factor receptor 1	Cediranib (VEGFR 1–3, PDGFR inhibitor); Sorafenib (VEGFR, PDGFR inhibitor); Sunitinib (VEGFR, PDGFR inhibitor); Nintedanib (VEGFR, PDGFR, FGFR inhibitor); Pazopanib (VEGFR, PDGFR, FGFR inhibitor); Lucitanib (VEGFR 1–3 and FGFR 1–2 inhibitor)	Ivy et al. [22]
sVEGFR-2	soluble vascular endothelial growth factor receptor 2		
SCF	stem cell factor	Imatinib (PDGFRs and SCF inhibitor); Sunitinib (VEGFR, PDGFR, SCF inhibitor)	Choi et al. [23]; Yasuda et al. [24]

**Table 4:** Study group characteristics.

Patient Group	Number of Samples (%)	Median Age (Min–Max)	Median BMI (Min–Max)	% of Postmenopausal
OC	38 (24.36)	60 (32–78)	25.12 (18.55–38.37)	79
Type I	7 (4.49)			
Type II	31 (19.87)			
Borderline	6 (3.85)	48 (37–52)	27.26 (17.29–31.64)	33
Benign ovarian tumour	62 (39.74)	40.5 (17–72)	24.31 (17.85–39.89)	26
Healty controls	50 (32.05)	56 (19–73)	25.80 (18.96–40.06)	60

**Table 5:** Characteristics of OC (ovarian cancer) group.

Histopathological Type	Number of Samples	Percentage (%)
Serous	16	42.11
Endometrioid	4	10.53
Mucinous	1	2.63
Clear cell	3	7.89
Undifferentiated	10	26.32
Non identified	4	10.53
FIGO stage at diagnosis		
I	10	26.32
II	2	5.26
III	25	65.79
IV	1	2.63

FIGO: International Federation of Gynaecology and Obstetrics.
